# Progress and challenges in engineering cyanobacteria as chassis for light‐driven biotechnology

**DOI:** 10.1111/1751-7915.13526

**Published:** 2019-12-27

**Authors:** Andrew Hitchcock, C. Neil Hunter, Daniel P. Canniffe

**Affiliations:** ^1^ Department of Molecular Biology and Biotechnology University of Sheffield Sheffield UK; ^2^ Institute of Integrative Biology University of Liverpool Liverpool UK

## Abstract

Cyanobacteria are prokaryotic phototrophs that, in addition to being excellent model organisms for studying photosynthesis, have tremendous potential for light‐driven synthetic biology and biotechnology. These versatile and resilient microorganisms harness the energy of sunlight to oxidise water, generating chemical energy (ATP) and reductant (NADPH) that can be used to drive sustainable synthesis of high‐value natural products in genetically modified strains. In this commentary article for the Synthetic Microbiology Caucus we discuss the great progress that has been made in engineering cyanobacterial hosts as microbial cell factories for solar‐powered biosynthesis. We focus on some of the main areas where the synthetic biology and metabolic engineering tools in cyanobacteria are not as advanced as those in more widely used heterotrophic chassis, and go on to highlight key improvements that we feel are required to unlock the full power of cyanobacteria for future green biotechnology.

## Graphical abstract

Cyanobacteria are promising chassis for light‐driven biotechnology. In this commentary for the Synthetic Microbiology Caucus we discuss the great progress that has been made in engineering these microorganisms for solar‐powered biosynthesis. We also give our opinion on key areas where the synthetic biology toolkit must be improved in order to unleash the full potential of cyanobacterial cell factories.

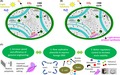

## Introduction

**Figure 1:**
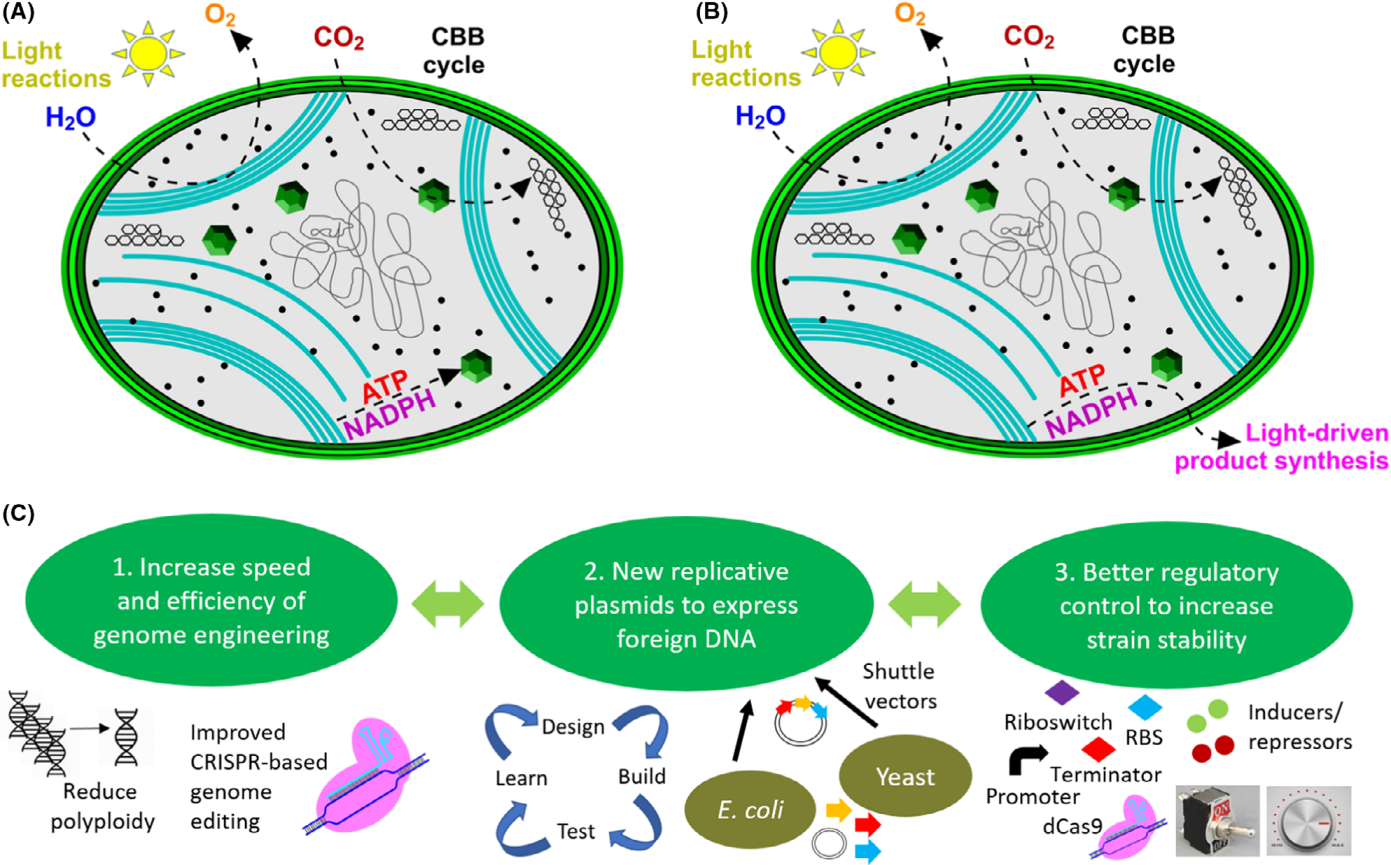
Engineering cyanobacterial chassis for light‐driven product synthesis. A. Schematic overview of a cyanobacterial ‘chassis’. The photosynthetic light reactions generate ATP and NADPH using solar energy and water. The ATP and NADPH are consumed by the Calvin–Benson–Bassham (CBB) cycle to convert atmospheric CO_2_ into carbohydrates. B. Genetic engineering approaches can be used to engineer cyanobacteria so that photosynthetically generated ATP and reductant can be used for light‐driven product synthesis. C. We suggest three overlapping and complementary areas for improvement that we believe will benefit the cyanobacterial synthetic biology community. (1) First, reducing polyploidy and/or improving CRISPR‐based genome editing would increase the speed, ease and efficiency of generating genetically modified strains. (2) New replicative shuttle plasmids for extra‐chromosomal expression of foreign genes and pathways are needed to enhance the ‘design‐build‐test‐learn’ cycle that lies at the heart of the synthetic biology process. (3) An improved ‘synthetic biology toolkit’ that can be used in a combinatorial fashion to allow precise temporal and orthogonal gene expression/protein production to improve product yields and minimize instability of engineered strains.

Cyanobacteria are a diverse group of prokaryotic microorganisms that inhabit a range of environmental niches. They perform oxygenic photosynthesis like plants and algae, utilizing solar energy and water to generate chemical energy (ATP) and reducing power (NADPH) to fix atmospheric carbon dioxide (CO_2_) to make carbohydrates (Fig. 1). Oxygen is a ‘waste’ product of this process and ancient cyanobacteria oxygenated the Earth’s atmosphere, permitting the evolution of complex, multicellular life forms. Cyanobacteria are excellent model organisms for fundamental photosynthesis research because of their relation to eukaryotic plastids, amenability to genetic engineering and relatively rapid generation time. They are also promising chassis for light‐powered biotechnological applications, requiring only sunlight, water, CO_2_ and trace inorganic minerals for growth, and tolerating variations in light, pH, salinity and temperature. Much progress has been made towards developing cyanobacteria as green microbial cell factories for the sustainable production of valuable natural products, chemicals and biofuels (see reviews by Oliver *et al.*, 2016; Khan *et al.*, 2019; Khan and Fu, 2019; Lin and Pakrasi, 2019); however, while the ‘synthetic biology toolkit’ for cyanobacterial metabolic engineering is advancing rapidly, it still lags behind those of model heterotrophic microorganisms (e.g. *Escherichia coli*) and yeast (*Saccharomyces cerevisiae*). Here, we consider the current situation with genetic engineering of cyanobacteria and highlight some key limitations that must be overcome in order to realize the full biotechnological potential of these versatile microorganisms.

## Modifying cyanobacterial genomes is simple but slow

For an organism to be a suitable chassis for metabolic engineering an annotated genome sequence should be available, and it must be amenable to genetic manipulation to allow efficient and rapid generation of knockout and ‘knock‐in’ strains. Many genetically tractable cyanobacteria meet these criteria (e.g. *Synechocystis* sp. PCC 6803, *Synechococcus* sp. PCC 7002, *Synechococcus elongatus* PCC 7942 and *Anabaena* sp. PCC 7120). Fast‐growing (e.g. *S. elongatus* UTEX 2973) and ‘non‐model’ species are also promising candidates for industrial applications (Yu *et al.*, 2015; Gale *et al.*, 2019; Mukherjee *et al.*, 2019). Suicide constructs for making targeted genomic modifications by double homologous recombination can be transferred into cyanobacteria by natural transformation, conjugation or electroporation. A number of ‘neutral’ chromosomal sites are available for integrating heterologous DNA, avoiding polar effects and undesirable changes in cell metabolism and physiology (Ng *et al.*, 2015; Pinto *et al.*, 2015). Some cyanobacteria also contain endogenous plasmids that can be used for expression of foreign genes (Xu *et al.*, 2011).

Marked genomic modifications are common in cyanobacteria as they allow positive selection and maintenance of engineered strains; however, only a limited number of antibiotic markers are available, restricting the number of sequential genetic manipulations that can be made. Furthermore, the metabolic load of multiple resistances can negatively affect cell performance, and antibiotic use is economically and ecologically undesirable in scaled‐up ‘real world’ applications. Strategies utilizing alternative phosphorus (phosphite) and nitrogen (melamine) sources for selection circumvent the antibiotic issue (Polyviou *et al.*, 2015; Selão *et al.*, 2019), but the problem of marker recycling remains. Marker‐less editing is therefore desirable and two‐stage selection/counter‐selection (Lea‐Smith *et al.*, 2016) and site‐specific recombinase (Tan *et al.*, 2013) systems can be used in cyanobacteria. However, because many cyanobacteria are relatively slow growing (compared with *E. coli*) and display polyploidy (multiple genome copies per cell), time‐consuming segregation to generate homozygous strains in which every copy of the genome has been modified slows down genetic engineering. This is doubly laborious for generating marker‐less strains, which require segregation after each transformation/recombination event. A better understanding of polyploidy may permit generation of organisms with only one genome copy per cell, which would speed up further genome modification but may come with an undesirable cost to cell fitness. The recently announced CyanoSource barcoded mutant library for the model species *Synechocystis* sp. PCC 6803 (Gale *et al.*, 2019) will be a valuable resource of knockout strains for the community.

## The potential of CRISPR genome editing in cyanobacteria

A better solution to the segregation time and multi‐loci/marker rescue problems is the use of clustered regularly interspaced short palindromic repeats (CRISPR) genome editing, which has been employed successfully in several cyanobacterial species (see Behler *et al.*, 2018 for a detailed review). CRISPR genome editing can target multiple genes in parallel, is precise, marker‐less and scar‐free, and can edit every copy of the chromosome with just one round of cell viability‐based selection. To date, the CRISPR‐machinery is introduced into cyanobacteria on an exogenous replicative plasmid (see below) that must be cured from the modified strain, slowing down the overall process. There are also issues with toxicity and ‘off‐target’ effects of the CRISPR nuclease and the efficiency of editing. These difficulties must be resolved for the true power of CRISPR genome editing in cyanobacteria to be unlocked, but it looks set to be an invaluable method for strain editing in the future.

## The need for replicative plasmids for propagation of exogenous DNA

While genome editing is essential in some circumstances, genes can also be introduced to cyanobacteria on self‐replicating plasmids, as is common with other synthetic biology chassis. Replicative plasmids overcome the time‐constraint of strain segregation and the difficulty of integrating larger inserts into the genome, and gene expression is often higher from plasmids than from the bacterial chromosome (Chen *et al.*, 2016). As discussed above, CRISPR‐ and recombinase‐based genome engineering also require expression of plasmid‐borne genes (Tan *et al.*, 2013; Wendt *et al.*, 2016). However, the use of plasmids in cyanobacterial genetic engineering is somewhat limited; only pRSF1010 derivatives or fusions of native cyanobacterial and *E. coli* plasmids have been widely used in cyanobacteria due to stability issues with other platforms (see Xia *et al.*, 2019 for an overview of replicative plasmids in cyanobacteria). A better understanding of cyanobacterial plasmid replication and the development of exogenous self‐replicating vectors based on alternatives to the RSF1010 backbone would be of great benefit to the cyanobacterial metabolic engineering community. Shuttle vectors that are maintained in both *E. coli*/ yeast and the cyanobacterial host are particularly useful as they facilitate high throughput assembly of constructs using modular DNA assembly methods in the former prior to transferring into the latter, speeding up the ‘design‐build‐test‐learn’ optimization cycle of synthetic biology.

## Genetic instability necessitates regulation of heterologous gene expression/protein production

Achieving specific and tuneable regulation of introduced genes and pathways is another significant hurdle in cyanobacterial metabolic engineering, and it can avoid uncontrolled expression of heterologous genes, and the accompanying negative effects on the cell (Jones, 2014). As cyanobacteria display adaptive evolution in response to stress, tight control of gene expression can prevent selection of faster‐growing loss of function suppressor mutations.

Control can be exerted at the transcriptional, translational and post‐translational levels, but well‐characterized regulatory elements used in other organisms do not tend to work as predicted when ported into cyanobacteria (Sengupta *et al.*, 2018). There has therefore been an enormous effort to develop and characterize genetic parts for cyanobacteria that can be used with standardized assembly methods such as the SyneBrick vectors (Kim *et al.*, 2017) and the CyanoGate modular cloning (MoClo) system (Vasudevan *et al.*, 2019). For example, promoters responding to isopropyl β‐D‐1‐thiogalactopyranoside (Huang *et al.*, 2010), anhydrotetracycline (Huang and Lindblad, 2013), sugars (Kelly *et al.*, 2018), light (Abe *et al.*, 2014), metals (Englund *et al.*, 2016), temperature (Mermet‐Bouvier and Chauvat, 1994) and metabolic/environmental signals (Immethun *et al.*, 2017) have been investigated, but in general their use is affected by poor dynamic range, leakiness, toxicity or photo‐lability of the inducer, and/or incompatibility with cyanobacterial growth conditions (Camsund and Lindblad, 2014). Native and synthetic promoters of different strengths can be used when constitutive gene expression is permitted/desired (Markley *et al.*, 2015; Ruffing *et al.*, 2016), although there may be unpredictable diurnal regulation as expression is often only tested under constant illumination.

Regulation of cyanobacterial transcription can also be achieved with riboswitches (Nakahira *et al.*, 2013; Ma *et al.*, 2014) or CRISPR interference (CRISPRi), which allows simultaneous repression of multiple genes (multiplexing) (Gordon *et al.*, 2016; Yao *et al.*, 2016). Other synthetic RNA‐based systems such as antisense repression and trans‐activating sRNAs are also effective in cyanobacteria (Ueno *et al.*, 2018). Transcriptional terminator libraries to prevent readthrough to downstream genes have been developed (Lui and Pakrasi, 2018), while at the post‐transcriptional level, ribosome binding site libraries for varying the strength of translation initiation (Wang *et al.*, 2018; Lui and Pakrasi, 2018) and degradation tags to control protein levels (Huang *et al.*, 2010; Landry *et al.*, 2013), have been described in cyanobacteria. A big challenge for the future is to combine this ever‐expanding range of genetic parts to achieve tight, dynamic, specific and orthogonal regulation of multiple components, allowing stable pathway maintenance and predictable performance under conditions relevant to scaled commercial applications.

## Concluding remarks: a bright, green future for cyanobacterial biotechnology

Great progress has been made in engineering cyanobacterial chassis as microbial cell factories for renewable and light‐driven synthesis of biochemicals and biofuels, but further improvements are now required to develop these processes into economically viable options for bioproduction. We suggest three main areas for improvement, summarized below and in Fig. 1. (i) Increase the speed and efficiency of marker‐less genome modification, for example by optimization of CRISPR‐based technologies. (ii) Development of new replicative shuttle plasmids for expression of heterologous genes and operons. (iii) Achieving tighter regulatory control of introduced pathways to increase long‐term strain stability. By combining such advances in the constantly developing cyanobacterial synthetic biology toolkit with knowledge from ‘omics’ and genome‐scale metabolic flux analyses/models (Hagerman and Hess, 2018; Hendry *et al.*, 2019), and new developments in the field of protein and pathway engineering, significant optimization of strain performance and product yield should be achievable. We are confident of a bright, green future for cyanobacterial biotechnology.

## Conflict of interest

None declared.
